# Does the Use of Proton Pump Inhibitors Increase the Risk of Pancreatic Cancer? A Systematic Review and Meta-Analysis of Epidemiologic Studies

**DOI:** 10.3390/cancers12082220

**Published:** 2020-08-08

**Authors:** Hee-Eun Hong, A-Sol Kim, Mi-Rae Kim, Hae-Jin Ko, Min Kyu Jung

**Affiliations:** 1 Department of Family Medicine, Kyungpook National University Hospital, Daegu 41944, Korea; hhe8824@naver.com (H.-E.H.); jmrtm@daum.net (M.-R.K.); liveforme@knu.ac.kr (H.-J.K.); 2 Department of Family Medicine, School of Medicine, Kyungpook National University, Daegu 41944, Korea; 3 Department of Family Medicine, Kyungpook National University Chilgok Hospital, Daegu 41404, Korea; 4 Department of Internal Medicine, School of Medicine, Kyungpook National University, Daegu 41944, Korea; minky1973@hanmail.net; 5 Division of Gastroenterology, Department of Internal Medicine, Kyungpook National University Hospital, Daegu 41944, Korea

**Keywords:** proton pump inhibitor, pancreatic cancer, pancreatic neoplasm, meta-analysis

## Abstract

*Background:* One of the most frequently used medications for treating gastrointestinal disorders is proton pump inhibitor (PPI), which reportedly has potential adverse effects. Although the relationship between the use of PPIs and the risk of pancreatic cancer has been extensively investigated, the results remain inconsistent. Hence, this meta-analysis aimed to evaluate such relationship. *Methods:* We searched for literature and subsequently included 10 studies (seven case–control and three cohort studies; 948,782 individuals). The pooled odds ratio (OR) and 95% confidence intervals (CI) for pancreatic cancer were estimated using a random-effects model. We also conducted sensitivity analysis and subgroup analysis. *Results:* The pooled OR of the meta-analysis was 1.698 (95% CI: 1.200–2.402, *p* = 0.003), with a substantial heterogeneity (I^2^ = 98.75%, *p* < 0.001). Even when studies were excluded one by one, the pooled OR remained statistically significant. According to the stratified subgroup analyses, PPI use, and pancreatic cancer incidence were positively associated, regardless of the study design, quality of study, country, and PPI type. *Conclusion:* PPI use may be associated with the increased risk of pancreatic cancer. Hence, caution is needed when using PPIs among patients with a high risk of pancreatic cancer.

## 1. Introduction

Pancreatic cancer is one of the deadliest malignancies, with a high mortality rate. According to the GLOBOCAN 2018 database, it is the seventh leading cause of cancer deaths in the world [1]. Moreover, it ranks fourth as a cause of cancer deaths in the United States, as reported by Cancer statistics, 2020 [2]. Despite having several therapies, the overall 5-year survival rate is approximately 9% [2,3].

Pancreatic cancer has many risk factors, including diabetes mellitus, chronic pancreatitis, and pancreatic cyst [4,5,6]. Several environmental risk factors, such as smoking, obesity, a “Western” dietary pattern (consumption of processed and smoked meat), *Helicobacter pylori*, and hepatitis B or C virus infection, were also reported [7,8,9,10,11,12,13,14].

Proton pump inhibitors (PPIs) are one of the most widely used medications for treating gastrointestinal disorders. Since their introduction into clinical practice in the late 1980s, PPIs have been approved for both acute and chronic management of several gastrointestinal diseases, such as peptic ulcer disease, gastroesophageal reflux disease, and Zollinger–Ellison syndrome. They are also effective in preventing nonsteroidal anti-inflammatory drug (NSAID)-associated gastroduodenal mucosal injury and eradicating *H. pylori* infection. Considering that PPIs are well tolerated and highly effective, their use has increased dramatically [15]. As their use increases, the concern about the risk of potential adverse effects also increases [16]. PPIs may increase the risk of *Clostridium difficile* infection in the colon [17]. Long-term PPI use may increase the risk of osteoporosis-related fractures of the hip, wrist, and spine [18]. PPIs’ inhibitive effects of gastric acid secretion affect mineral bioavailability, resulting in the decreased intestinal absorption of calcium, magnesium, and iron [19,20,21]. Prolonged PPI use also reduces vitamin B12 (cyanocobalamin) absorption [22]. Moreover, studies of patients with long-term PPI medication showed an increased risk of heart attack [23]. The association between PPI use and the risk of pneumonia, kidney disease, and dementia has also been identified [24].

Indeed, the use of PPIs is associated with the increased risk of gastric cancer and hepatocellular carcinoma [25,26,27,28]. An association between PPI use and the risk of colorectal cancer was also reported [29]. Given that PPI use is associated with the increased risk of several cancers, the carcinogenic effects of PPIs have been investigated. In addition, PPI use is associated with the potential increased risk of pancreatic cancer [30].

However, only few studies investigating the association between PPI use and risk of pancreatic cancer have been published, reporting equivocal results. Therefore, in this meta-analysis, we aimed to investigate the association between PPI use and the risk of pancreatic cancer.

## 2. Materials and Methods

### 2.1. Search Strategy

We conducted a literature search to retrieve articles concerning the association between PPI use and the risk of pancreatic cancer between November 2018 and April 2020 (last date searched). We searched in the following four databases: PubMed, SCOPUS, Cochrane library and Google scholar. The keywords used for literature search were: “proton pump inhibitor” OR “proton pump inhibitors” OR “PPI” OR “PPIs” AND “pancreatic cancer” OR “pancreatic neoplasm” OR “pancreatic ductal adenocarcinoma” OR “pancreatic adenocarcinoma”.

Following the inclusion and exclusion criteria, three authors (HEH, ASK, and MRK) independently searched the literature and extracted eligible articles. Articles written only in English were selected, while the duplicated articles were eliminated. After the irrelevant articles were excluded by screening the titles and abstracts, the full-text articles of the remaining articles were reviewed, and the required information was then collected. Thereafter, we exported the results to a reference manager (EndNote) file. In the case of discrepancy, we consulted another author (HJK) and determined the final decision by mutual discussion. To identify any other relevant articles, we also searched the references of each article. This meta-analysis conformed to the Preferred Reporting Items for Systematic Reviews and Meta-Analyses (PRISMA) statement [31].

### 2.2. Study Selection

The inclusion criteria were as follows: (1) human studies; (2) case–control or retrospective cohort studies; (3) studies reporting or containing the association between PPI use and the risk of pancreatic cancer; (4) studies reporting the incidence or prevalence of pancreatic cancer; (5) studies providing available data to calculate the odds ratio (OR) or hazard ratio (HR) with 95% confidence intervals (CI), with P values; and (6) articles written in English.

Conversely, the exclusion criteria were as follows: (1) duplicated articles; (2) abstracts, case reports, comments, and reviews; (3) in vivo studies (animal study) and in vitro studies (experimental study designs in laboratory settings); (4) studies regarding the mortality or survival rate of pancreatic cancer in patients who had used PPIs; (5) other languages; and (6) studies without relevant data.

### 2.3. Data Extraction

For the meta-analysis, we extracted data regarding the PPI exposure and pancreatic cancer incidence from each study. We double-checked all studies and resolved the differences by discussion and consensus. For each study, the following information was recorded: publication details (including the name of the first author and the year of publication), country of publication, study design, characteristics of the studied population (including mean age and ratio of sex), outcome (including OR and 95% CI), the diagnostic criteria of pancreatic cancer, and the definition of PPI exposure.

For the early undiagnosed pancreatic cancer symptoms that often present as nonspecific abdominal symptoms, PPIs may have been administered to alleviate these symptoms. Hence, to reduce protopathic bias, we excluded pancreatic cancer cases that were diagnosed within 1 or 2 years after PPI exposure in six of the selected articles [30,32,33,34,35,36]. In agreement with this decision, we extracted the data of long-term users in Kearns et al. [37].

### 2.4. Quality Assessment

Using the Newcastle–Ottawa Scale (NOS) [38], we assessed the methodological quality of the studies. The NOS contains eight items, which are categorized into the following three parts: selection, comparability, and outcome (on cohort studies) or exposure (on case–control studies). The score of NOS ranges from 0 to 9 stars. NOS has no definite cutoff value that defines a high-quality study. In this meta-analysis, the mean value of the selected studies was 7.1. Therefore, we defined >7 stars as a high-quality study in this analysis.

### 2.5. Statistical Analysis

Using the DerSimonian–Laird method, we employed the random-effects model to estimate the summary OR and 95% CI [39], which were both calculated to assess the risk of pancreatic cancer from PPI exposure. The heterogeneity among the studies was assessed using the *p* value of the χ^2^-based Cochrane Q tests and the inconsistency score (I^2^). The heterogeneity test addressed the null hypothesis that all studies in the meta-analysis share a common effect size. A significant χ^2^ test result (*p* < 0.05) indicated a significant heterogeneity. The high, moderate, low, and no heterogeneity corresponded to the I^2^ values of ≥75, 50–74, 25–49, and <25%, respectively. Potential publication bias was evaluated using Begg’s funnel plot and Egger’s regression test [40]. Begg’s funnel plot is a scatter plot with an effect size on the horizontal axis and the sample size or variance on the vertical axis. To detect publication bias, we checked the asymmetry of distribution in the funnel plot displaying the relationship between study size and effect size. Meanwhile, the degree of asymmetry was assessed by Egger’s test; *p* < 0.05 indicated a statistically significant publication bias. All analyses were conducted using the Comprehensive Meta-Analysis version 2.2.064, and the statistically significant level was 0.05.

## 3. Results

### 3.1. Selected Studies

**Figure 1:**
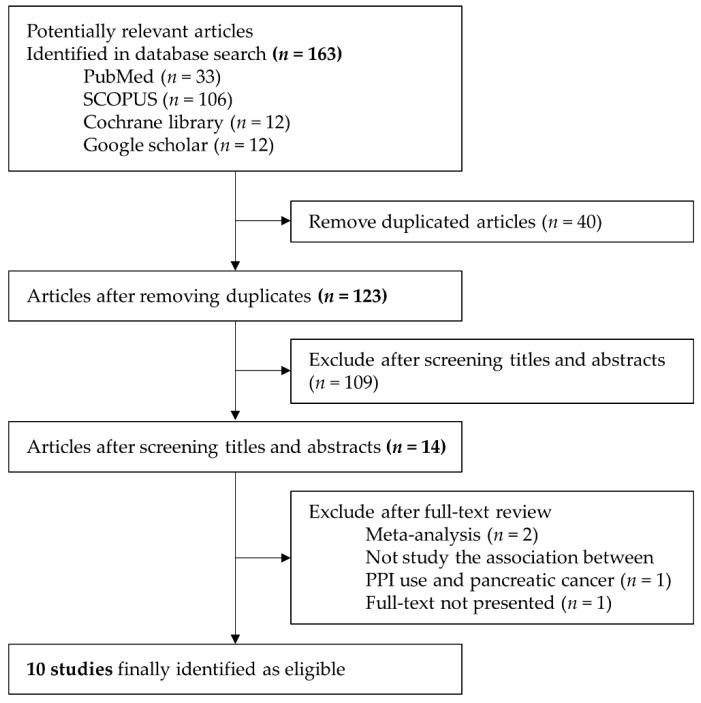
Flow diagram of the literature search and selection of studies for the meta-analysis.

Through database search and the reference list, we identified the first 163 potentially relevant articles: PubMed, *n* = 33; SCOPUS, *n* = 106; Cochrane library, *n* = 12; and Google scholar, *n* = 12. After removing the duplicates, we identified 123 studies. After screening the titles and abstracts, we excluded 109 articles because they were considered irrelevant articles. After performing a full-text review of the 14 remaining articles, we excluded one article which had no full-text; two articles which were meta-analysis; and one article which had a different outcome. Ultimately, 10 studies met the inclusion criteria, and thereby were considered as eligible. Figure 1 presents a flow chart of the literature identification process.

Of these 10 studies, seven were case–control studies, and three were cohort studies. They were published between 2012 and 2020 in six countries. Three studies were conducted in the UK [36,37,41], two in the USA [32,42], two in Taiwan [30,33], one in Korea [35], one in Denmark [34] and one in Sweden [43]. The follow-up duration was between 5 and 20 years. The meta-analysis then included 948,782 subjects. All studies contained both men and women, and the mean age ranged from 57.3 years to 71.1 years. All selected studies obtained results that indicate an association between PPI exposure and pancreatic cancer incidence. The diagnosis of pancreatic cancer was based on the ICD codes in six studies [30,32,33,35,42,43], medical record code in two studies [36,37], and histological verification of PDA in two studies [34,41]. Stratified analysis according to the PPI dose and duration was performed in five studies [32,33,34,35,36] and in the five other studies [32,33,36,37,43], respectively, although they all used a different cutoff value of dose and duration. In six studies, pancreatic cancer cases that developed within 1 or 2 years after PPI introduction were excluded because PPI was probably used to help in treating and diagnosing cancer-associated symptoms [30,32,33,34,35,36]. Table 1 lists the clinical characteristics of the included studies.

### 3.2. PPI Use and the Risk of Pancreatic Cancer

**Figure 2:**
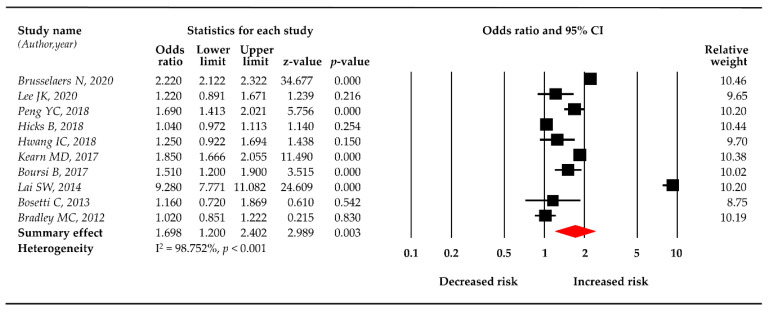
Forest plot of the association of proton pump inhibitors (PPIs) and the risk of pancreatic cancer.

Using the random-effects model, the meta-analysis of 10 studies revealed that PPI use was significantly related to pancreatic cancer risk (OR = 1.698, 95% CI: 1.200–2.402). The log odds ratio also showed that PPI use had a significant association with the risk pancreatic cancer (log OR = 0.529, 95% CI: 0.182–0.876), with a substantial heterogeneity (I^2^ = 98.75%, *p* < 0.001). Figure 2 illustrates the main result of the meta-analysis using the random-effects model.

### 3.3. Sensitivity Analysis

To estimate the accuracy and robustness of the pooled effect size, we conducted sensitivity analysis by excluding each study one by one. According to the pooled OR of each analysis, PPI use was positively associated with the risk of pancreatic cancer, demonstrating statistical significance (Table 2).

### 3.4. Subgroup Analysis

Subsequently, a series of subgroup analyses that examined the robustness of the result and explored potential sources of heterogeneity were conducted according to the study characteristics such as study design, quality of study, counties of publication, and PPI type. A positive association was consistently observed, regardless of these characteristics (Table 3).

As mentioned, seven and three of the 10 studies were case–control and cohort studies. According to the study design, the summary effect of case–control studies was significant, with an OR of 1.725 (95% CI: 1.005–2.959, *p* = 0.048) compared with 1.647 (95% CI: 1.134–2.392, *p* = 0.009) of the cohort studies.

In this meta-analysis, studies with seven to nine stars are considered high quality (*n* = 4), whereas those with <6 stars are considered low quality (n = 6). With stratification by the quality of study, PPI use demonstrated a statistically significant positive association with the risk of pancreatic cancer, irrespective of the scale of quality. The effect size was 1.534 (95% CI: 1.081–2.176, *p* = 0.017) in high-quality studies and 1.824 (95% CI: 1.005–3.312, *p* = 0.048) in low-quality studies.

We also conducted a subgroup analysis by countries of publication. Of the 10 studies, three were published in Asian countries (Taiwan and Korea), and seven were published in Western countries (Europe and USA). With stratification by countries, both groups demonstrated a positive association between PPI exposure and pancreatic cancer incidence. The summary effect OR for the subgroup analysis of studies in Asian countries was 2.705 (95% CI: 0.751–9.746), whereas that in Western countries was 1.388 (95% CI: 0.996–1.934).

According to PPI type, three of the 10 studies showed stratified results. The PPIs used in the studies were omeprazole, pantoprazole, lansoprazole, rabeprazole, and esomeprazole. This subgroup analysis showed the significant association between PPI use and pancreatic cancer risk. The OR of pancreatic cancer was 2.113 (95% CI: 0.697–6.411), 2.524 (95% CI: 0.484–13.156), 2.985 (95% CI: 0.771–11.556), 5.401 (95% CI: 1.984–14.703), and 2.583 (95% CI: 0.475–14.056) on omeprazole, pantoprazole, lansoprazole, rabeprazole, and esomeprazole, respectively.

To clarify the association between PPI exposure and pancreatic cancer risk, we analyzed the dose–response and duration–response data. Of the 10 studies, five showed dose–response results, while the other five showed duration–response results. However, each study presented a different cutoff value of dose and duration. Therefore, we could not perform the subgroup analysis for both dose and duration of PPI.

### 3.5. Publication Bias

**Figure 3:**
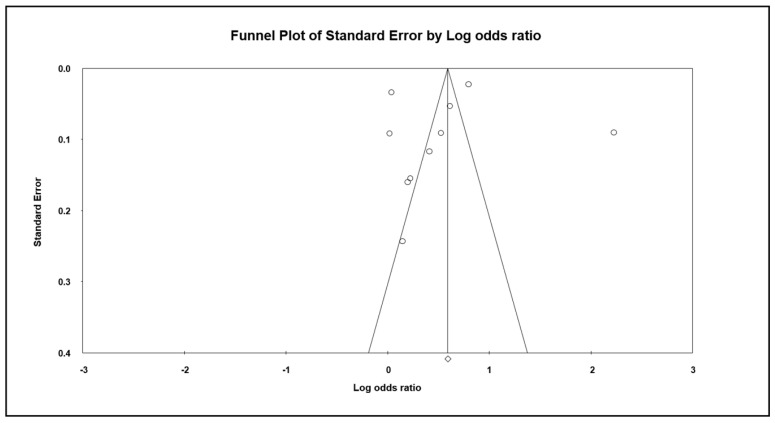
Funnel plot and Egger’s regression intercept.

To detect publication bias, we employed the Egger’s regression and created the Begg’s funnel plots. As showed in Figure 3, the distribution of studies on both sides was relatively symmetric, indicating that our meta-analysis had no possible bias (*p* = 0.840). When four studies were added using Duval and Tweedie’s trim-and-fill methods, the adjusted OR was 2.243 (95% CI: 2.179–2.308); hence, the impact of this bias was probably trivial.

## 4. Discussion

This meta-analysis aimed to elucidate that the use of PPIs, one of the widely used medications for treating gastrointestinal disorders, has a possibility to increase the risk of pancreatic cancer. This study is meaningful in concluding the results of the prior observational studies. It analyzed 10 studies, which included a total of 948,782 patients, by using a random-effects model and showed that PPI exposure was associated with a high pancreatic cancer risk of 69.8%.

This result could be supported by some theories whereby the PPIs may influence pancreatic cancer [44]. Indeed, several mechanisms have suggested that PPIs have potential carcinogenic effects in pancreatic cancer. Such effects include the increased production of gastrin and the effects of gastric hypoacidity on microbes.

### 4.1. Increased Production of Gastrin

The first mechanism—that is, increased gastrin production—has a carcinogenic effect on pancreatic cancer pathophysiology. Gastrin is produced in neuroendocrine G cell in the antrum and acts physiologically as a hormone to stimulate acid secretion. As the released gastrin binds to CCK2 receptors on enterochromaffin-like (ECL) cells, ECL cells secrete histamine, which binds to H2 receptor on parietal cells and stimulates acid secretion [45]. Furthermore, the gastrin–CCK receptor stimulates gastric epithelial cell migration, fibroblast growth factor release, and protein kinase pathway activation [46]. Gastrin is associated with the development and progression of gastrointestinal malignancies, including gastric cancer and colorectal cancer [47,48]. Indeed, gastrin is related to pancreatic cancer tumorigenesis. PPIs inactivate the H+/K+ ATPase (proton pump) on parietal cells in the stomach; thus, gastric acid secretion is reduced. Gastric acid suppression creates a strong stimulus for gastrin production in G cells, resulting in hypergastrinemia [49]. In addition, human pancreatic cancer cells express gastrin receptors [50]. Through the gastrin receptor, gastrin stimulates the growth of human pancreatic cancer cells [51,52,53], as shown in cultures and tumors transplanted in nude mice [51]. Moreover, the following also supports the effects of gastrin as a growth factor for pancreatic cancer. The specific gastrin-receptor antagonist L-365,260 blocks the stimulation of cell replication by gastrin, thereby inhibiting pancreatic cancer cell growth [51]. Additionally, the gastrin-receptor antagonist gastrazole (JB95008) increased the survival time of pancreatic cancer compared with placebo [54]. In patients with pancreatic cancer, responders to gastrin-17-diphtheria toxoid immunogen (G17DT), which is an anti-gastrin antibody, showed a significantly longer survival than antibody non-responders [55,56].

As gastrin binds to CCK-2 receptor, the stimulated CCK-2 receptor activates several kinases and signaling pathways that are related to pancreatic adenocarcinoma upregulation [57]; some of them are the JAK2/STAT3 pathway [58], Src-related tyrosine kinases, and p125FAK, which play a crucial role in gastrin effects [59]. Recently, alphaV integrin, which is a new gastrin target gene in human pancreatic cancer cells, has been identified [60].

### 4.2. Bacterial Overgrowth and Nitrosamine

The second mechanism is that microbe overgrowth according to gastric hypoacidity induces nitrosation. The association between nitrosamine and cancer was previously investigated, particularly in the cancer of the stomach, esophagus, nasopharynx, urinary bladder, and colon [61,62]. Exposure to N-nitroso compounds (NOCs) or endogenous formation produces nitrosamine. One may be exposed to NOCs through diet, certain occupational exposure, tobacco products, cosmetics, pharmaceutical products, and agricultural chemicals. Nitrosamine could also be produced endogenously through acid-catalyzed N-nitrosation at acidic gastric conditions (<pH 2.5) or through bacterially catalyzed N-nitrosation at gastric pH increases from 5 to 8 [63,64]. Moreover, nitrosamine delivered by smoking and dietary sources, especially smoked and processed meat, has carcinogenic effects in pancreatic cancer [11,65]. Gastric hypoacidity favors the overgrowth of nitrate-reducing bacteria in the stomach [66]. The nitrate-reducing bacteria convert nitrate into nitrite, leading to the reduction of luminal nitrate and the rapid production of nitrite. Faster nitrosation of luminal amines triggers the production of potentially carcinogenic N-nitrosamines in the lumen [66]. Pancreatic ductal adenocarcinomas have been found in nitrosamine-exposed mice and human pancreatic cells (in vitro) [67]. N-nitrosamine carcinogens attribute DNA damage by forming methyl and 2-hydroxypropyl adducts. Indeed, they are linked to the synthesis and replication of adduct-bearing DNA, particularly in the pancreatic ductular epithelium [68,69].

Furthermore, secretin acts as a carcinogen of pancreatic cancer. PPIs could induce the increase in secretin levels, consequently affecting pancreatic cell growth. Chronic secretin stimulation and NOC exposure potentially overwhelm DNA repair capabilities, acting synergistically to induce tumor development [70].

Histamine-2-receptor antagonist (H2RA), an anti-acid agent similar to PPIs, induces hypergastrinemia and hypoacidity; hence, H2RA is also reportedly associated with pancreatic cancer; the studies are still underway [36].

### 4.3. The Biological Link Between PPI Use and Other Cancers

These mechanisms suggesting a biological link between PPI use and cancer risk have been found in other cancers [71]. In addition, gastrin and cancer correlation has been demonstrated in gastric, colorectal, and hepatocellular cancers [47,72,73,74,75]. Gastrin is associated with gastric cancer proliferation by an autocrine mechanism [73]. It is also expressed in liver tumor; this expression may be associated with tumor proliferation [74]. Indeed, tumor induction by endogenously formed N-nitrosamine is also found in these cancers [29,76]. These findings support the carcinogenic effects of PPIs, and further studies are required to investigate this association.

### 4.4. Data Interpretation

In this meta-analysis, the significant association of PPI use and pancreatic cancer risk was maintained in the sensitivity analysis, which excluded each study separately. In addition, the effect size in the subgroup analysis based on the type of study design, quality of study, and country of publication was also statistically significant. Interestingly, in the subgroup analysis based on PPI type, only rabeprazole showed a significant association between PPI use and pancreatic cancer risk. The reason could be that the number of studies (two studies) concerning rabeprazole was less than that in other PPI types, both studies were conducted in the same country, and one of these studies in particular had a very high OR. Otherwise, the peculiar pharmacodynamics that rabeprazole has the highest pKa could explain this result. The pKa is the pH at which 50% of the drug becomes ionized (protonated). According to the Henderson–Hasselbalch equation, the amount of drug ionized form, which cannot cross cell membranes and results in drug accumulation, depends on the pKa at a given pH. The pKa of the PPIs ranges from approximately 4.0 for omeprazole to around 5.0 for rabeprazole. Therefore, at any given pH, rabeprazole accumulation within the parietal cells would be approximately tenfold that for omeprazole. Given its higher pKa, rabeprazole is activated over a wider pH range; consequently, it is converted into the active metabolite more efficiently. Their accumulation in the parietal cell or acid-induced activation of the PPI prodrug contributes to faster onset and greater gastric acid suppression compared with the other PPIs [77,78].

### 4.5. Limitations

However, this meta-analysis has several limitations. First, each study was inconsistent in defining the dose and duration of PPIs. Regarding the dose, one study used “pills/day” [32], whereas four studies used “defined daily dose (DDD)” as a unit [33,34,35,36]. Moreover, the cutoff value of dose was different in each study. For instance, Peng et al. used <30, 30–65, 65–150, and >150 DDD [33], whereas Hicks et al. used 1–99, 100–499, 500–999, 1000–2000, and >2000 DDD [34], as cutoff values. Considering the carcinogenic effect of PPIs on cancer, the risk of pancreatic cancer could be increased when using high-dose or long-term PPIs. If with some uniform measurement, we could conduct the dose- or duration-response analysis to evaluate the linear relationship, which helps quantify the association. Second, a significant heterogeneity was found in this meta-analysis. Moreover, the inconsistent cutoff value of dose and duration that we mentioned above could contribute to this heterogeneity. Hence, we used a random-effects model that considers the average effect size as the estimated mean value of the distribution of effect sizes for heterogeneous populations. Third, this meta-analysis included only 10 studies for the final analysis, thus the statistical power might not be enough to draw a definite conclusion. Fourth, this meta-analysis included only studies published in English, and small studies with cumulative results tend not to be published, resulting in potential bias.

Despite these limitations, this meta-analysis is meaningful for assessing the effect of PPIs, which are commonly used in clinical trials, on its relationship with pancreatic cancer with high mortality.

## 5. Conclusions

In conclusion, PPI use may be associated with the increased risk of pancreatic cancer. Therefore, medical professionals should carefully consider the prescription of PPIs for patients with a high risk of pancreatic cancer.

## Figures and Tables

**Table 1:** Baseline characteristics of included studies.

Study (*Author, Year*)	Study Design	Country	Period of Recruitment	No. of Study Population (Case/Control)	Mean Age (Years) (Case/Control)	Percentage of Males (Case/Control)	Confounder Adjusted in the Multivariate Analysis	Quality Assessment (NOS)
*Brusselaers N, * *2020*	Cohort	Sweden	2005–2012	796492	NA	41.5	Age, indications for gastric acid suppressive therapy, diabetes	8
*Lee JK, * *2020*	Case–control	USA	1996–2016	567/4870	67.8/67.3	50.6/ 51.5	Chronic alcohol consumption, smoking, BMI, family history of each cancer, cystic fibrosis, chronic pancreatitis, diabetes mellitus, pancreatic cysts	8
*Peng YC, * *2018*	Case–control	Taiwan	2006–2011	1087/1087	68.3/67.4	60.9/59.8	Age, chronic pancreatitis, biliary tract disease	6
*Hicks B, * *2018*	Case–control	Denmark	2000–2015	6921/34605	NA	NA	Diabetes, alcohol-related disease, COPD, chronic pancreatitis, gallstones, peptic ulcer, Helicobacter pylori infection, hepatitis B and C infection, use of low-dose aspirin, NSAIDs, statins, HRT, CCI, highest achieved education	7
*Hwang IC, * *2018*	Cohort	Korea	2002–2013	453655	NA	53.5	Age, BMI, smoking, alcohol, drinking, physical activity, diabetes, chronic pancreatitis, CCI, SES	9
*Kearn MD, * *2017*	Nested case–control, Cohort	UK	1995–2013	4113/16072	70.9/71.1	51.4/51.1	Diabetes, smoking, alcohol, obesity	6
*Boursi B, * *2017*	Cohort	UK	1995–2013	19146	62.7	53.6	NA	9
*Lai SW, * *2014*	Case–control	Taiwan	2000–2010	977/3908	68.38/68.11	60.59/60.59	Acute pancreatitis, chronic pancreatitis, diabetes, obesity, H2RA, statin, non-statin lipid lowering, both ASA and COX2i	6
*Bosetti C,* *2013*	Case–control	USA, Canada, Australia		56/51	NA	56.5/56.6	NA	5
*Bradley MC, * *2012*	Case–control	UK	1995–2006	1141/7954	57.3	533.7	Smoking, BMI, alcohol, history of chronic pancreatitis, use of other drugs (NSAIDs, steroids, HRT), diabetes, prior cancer	7

Abbreviations: NOS, Newcastle–Ottawa scale; BMI, body mass index; COPD, chronic obstruction pulmonary disease; HRT, hormone replacement therapy; CCI, Charlson comorbidity index; SES, socioeconomic status; NA, not applicable; H2RA, histamine-2 receptor antagonists; ASA; aspirin; COX2i; cyclooxygenase-2 inhibitor.

**Table 2:** Sensitivity analysis by excluding each study one-by-one.

Excluded Study *(Author,year)*	Observed OR	Effect Size and 95% Confidence Interval			Test of Null (Two-Tailed)	
		Mean OR without This Study	Lower Limit	Upper Limit	*z*-Value	*p-*Value
*Brusselaers N, 2020*	2.220	1.641	1.049	2.567	2.171	0.030
*Lee JK, 2020*	1.220	1.759	1.217	2.540	3.008	0.003
*Peng YC, 2018*	1.690	1.698	1.163	2.479	2.740	0.006
*Hicks B, 2018*	1.040	1.754	1.214	2.535	2.992	0.003
*Hwang IC, 2018*	1.250	1.800	1.273	2.545	3.328	0.001
*Kearn MD, 2017*	1.850	1.678	1.120	2.516	2.507	0.012
*Boursi B, 2017*	1.510	1.720	1.184	2.497	2.849	0.004
*Lai SW, 2014*	9.280	1.405	1.062	1.859	2.379	0.017
*Bosetti C, 2013*	1.160	1.761	2.223	2.535	3.043	0.002
*Bradley MC, 2012*	1.020	1.799	1.245	2.600	3.125	0.002

**Table 3:** Subgroup analysis according to study design, quality, country, and type of drugs.

Subgroup	No. of Studies	Effect Size and 95% Confidence Interval			Test of Null (Two-Tailed)	
		OR	Lower Limit	Upper Limit	*z*-Value	*p*-Value
**Study design**						
Case–control	7	1.725	1.005	2.959	1.978	0.048
Cohort	3	1.647	1.134	2.392	2.620	0.009
**Quality of study**						
High (NOS > 7)	4	1.534	1.081	2.176	2.394	0.017
Low (NOS ≤ 7)	6	1.824	1.005	3.312	1.975	0.048
**Countries**						
Asia	3	2.705	0.751	9.746	1.522	0.128
Western	7	1.388	0.996	1.934	1.934	0.053
**Type of drugs**						
Omeprazole	3	2.113	0.697	6.411	1.322	0.186
Pantoprazole	3	2.524	0.484	13.156	1.099	0.272
Lansoprazole	3	2.985	0.771	11.556	1.584	0.113
Rabeprazole	2	5.401	1.984	14.703	3.301	0.001
Esomeprazole	3	2.583	0.475	14.056	1.098	0.272
